# Australian Society of Medical Imaging and Radiation Therapy Image Registration in Radiation Therapy Position Paper

**DOI:** 10.1002/jmrs.70071

**Published:** 2026-02-18

**Authors:** Tamara Barry, Huong Nguyen, Laurel Schmidt, Kate Stewart, Nigel Anderson

**Affiliations:** ^1^ Australian Society of Medical Imaging and Radiation Therapy Image Registration in Radiation Therapy Working Group Melbourne Victoria Australia; ^2^ Department of Radiation Oncology Princess Alexandra Hospital, Metro South Health Woolloongabba Queensland Australia; ^3^ Icon Cancer Centre South Brisbane Queensland Australia; ^4^ Department of Radiation Oncology St George Cancer Care Centre, St George Hospital Kogarah New South Wales Australia; ^5^ Department of Radiation Oncology Royal Brisbane and Women's Hospital Herston Queensland Australia

**Keywords:** deformable image registration, guideline, image registration, radiation therapy, rigid image registration

## Abstract

The report from the American Association of Physicists in Medicine (AAPM) Task Group 132, published in 2017, established a framework and recommendations for the safe implementation of rigid image registration (RIR) and deformable image registration (DIR) into radiation oncology clinical practice. The Medical Image Registration Special Interest Group (MRSIG) of the Australasian College of Physical Scientists and Engineers in Medicine further built on these recommendations through the publishing of best practice guidelines for DIR warping based on increased accessibility to such tools in the clinical environment. There remains an increasing responsibility on radiation therapists, a critical member of the radiation oncology multidisciplinary team, to safely embed RIR and DIR process into their routine clinical practice, along all steps of the patient's radiation therapy treatment journey. This position paper, authored by the Australian Society of Medical Imaging and Radiation Therapy (ASMIRT) Image Registration Working Party and endorsed by the ASMIRT Executive, (i) details the role of radiation therapists in the application of RIR and DIR in day‐to‐day clinical practice and (ii) delivers a series of recommendations to support the safe implementation of RIR and DIR into radiation therapist workflows.

## Graphical abstract

The ASMIRT Image Registration Working Party outlines the critical role of radiation therapists in performing, assessing and documenting rigid and deformable image registration across treatment planning, delivery and adaptive workflows. The position paper emphasises consistent nomenclature, robust QA processes, strong collaboration with diagnostic imaging and the need for RT credentialing to ensure safe and accurate use of IR technologies. Furthermore, the paper provides a series of recommendations to standardise practice and support high‐quality, patient‐centred radiation therapy in Australia.

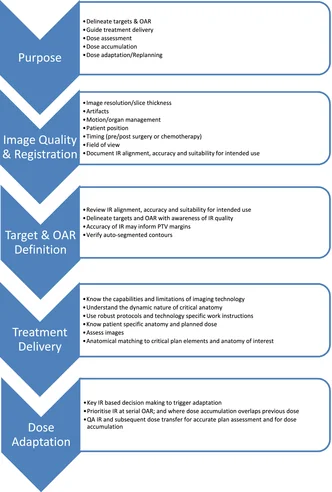

**Abbreviations**
IR: image registration
OAR: organs at risk
PTV: planning target volume
QA: quality assurance

## 1 Introduction

Robust, reliable and geometrically accurate imaging is a fundamental element of radiation therapy, resulting in image registration (IR) being well established in the daily practice of radiation therapists (RTs) in both dosimetry and treatment roles [1]. Multiple co‐registered images are frequently used together with the routinely acquired planning computed tomography (CT) scans for target volume and normal tissue delineation. These include, but are not limited to, CT, magnetic resonance imaging (MRI) and positron emission tomography (PET). These co‐registered datasets assist contour propagation and dose calculation, which occurs prior to the IR to guide sub‐millimetre and sub‐degree accuracy in treatment delivery. Subsequent daily dose assessment, dose warping or adaptation, and dose accumulation to evaluate the impact of anatomical changes on delivered dose also relies on registration of multiple image sets. These functions comprise standard day‐to‐day care in some circumstances (e.g., online adaptive MRI and CT‐based platforms) and are reliant on quality image acquisition, registration and application.

The number of imaging data sets used through a patient's radiation therapy journey has increased significantly as planning, treatment and re‐treatment has become more sophisticated. IR is also crucial in adaptive and replanning workflows, which have become routine daily practice. In Queensland, 21% of patients receive more than one course of RT during their cancer journey [2]. It is common for patients to receive care in different departments such that data management and sharing may be critical to optimal patient care.

Rigid image registration (RIR) functionality is a standard inclusion in treatment planning, treatment delivery and Oncology Information Systems (OIS), with deformable image registration (DIR) functionality readily available in most treatment planning or ancillary systems. Uptake of IR has occurred at different rates around the world due to funding and access to useful imaging modalities and technology [3]. For example, access to prostate‐specific membrane antigen (PSMA) positron emission tomography (PET), while available in Australia from 2016, was restricted in North America until December 2020 [4]. Similarly, access to MRI can be hampered by wait lists and relatively few departments thus far have been able to install dedicated MRI Simulators in Australia to allow reliable access for radiation therapy planning purposes.

Given the disparity of imaging availability and technological resourcing, clinics have adapted as resources become available, therefore position papers and robust consensus or quality assurance (QA) guidelines have followed widespread clinical use of IR practice [5, 6, 7, 8, 9]. Hence, use of IR throughout radiation therapy planning and treatment practice is largely reliant on the expertise and experience of the clinicians performing those activities daily in clinics. Although fundamental to capturing the essence of many aspects of IR and image guided radiation therapy (IGRT), each of these documents presents an international perspective that does not truly capture the unique clinical workflows of an Australian radiation oncology department, and more specifically, the role of an Australian radiation therapist. This necessitates the need for local consensus documentation to guide IR practice.

In Australia, the responsibility for retrieval, import and registration of secondary images for target and organ at risk (OAR) delineation rests largely with RTs. Similarly, through IGRT at treatment delivery, RTs lead this role, in some cases with decision support or oversight from Radiation Oncologists (ROs). IR requires a fundamental understanding of anatomical processes and clinical pathology, and the relationship of these to individualised dosimetry for each patient case.

RTs perform critical roles through many steps in the patient pathway where IR is required, and to impact, inform and advocate on behalf of RTs and patients in the rapidly evolving IR space. This position paper, authored by the Australian Society of Medical Imaging and Radiation Therapy (ASMIRT) Image Registration Working Party and endorsed by the ASMIRT Executive, (i) details the role of RTs in the delivery of RIR and DIR in day‐to‐day clinical practice and (ii) delivers a series of recommendations to support the safe implementation of RIR and DIR into RT workflows.

## 2 ASMIRT Position on Data Management Considerations for IR

### 2.1 Digital Imaging and Communications in Medicine (DICOM) Dataset Access

Cancer diagnosis normally involves multiple modality imaging studies that are frequently used in the radiation therapy planning process to aid in target delineation and subsequent treatment response assessment. Access to diagnostic imaging studies is critical to timely care, therefore, it is essential that departments are readily able to retrieve imaging required for treatment planning from Picture Archiving and Communication Systems (PACS) in their local environment. Additionally, establishing relationships between RTs and their Medical Imaging counterparts in Diagnostic Radiology Departments which scan their patients can be beneficial to the acquisition of improved imaging for RT purposes, for example, scanning patients in radiation therapy treatment position. Furthermore, there is a body of growing clinical evidence that presents the use of diagnostic scans alone being used for radiotherapy planning, that is, simulation‐free planning [10, 11, 12, 13]. To minimise data storage requirements, clear communication with the treating radiation oncologist is recommended to ensure that only the necessary dataset sequences are imported into the treatment planning system (TPS).

### 2.2 Nomenclature

Naming of image datasets should follow a clear system, for example, Trans‐Tasman Radiation Oncology Group (TROG) Standardised Naming and Contouring Guidelines [14]. This is consistent with wider global practice, where documents such as AAPM TG‐263 have long been developed and implemented, and form the foundation for TROG guidelines [15]. Where IR is introduced, the Medical Image Registration Special Interest Group (MIRSIG) as part of the Australasian College of Physical Scientists and Engineers in Medicine (ACPSEM) agree for naming to first identify the type of registration (RIR or DIR) followed by the name of the primary dataset (usually the planning CT scan) then the secondary dataset name [16]. Our recommendation is to align with this practice to ensure consistency across disciplines working in the same clinical environment with equivalent datasets.

## 3 ASMIRT Position on QA Considerations of IR

### 3.1 Image Quality and Registration Uncertainties

Diagnostic imaging studies that are used for radiation therapy treatment planning often are performed pre‐surgery or chemotherapy, and not in treatment position, adding complexity to IR with the planning CT scan. For example, a diagnostic imaging CT performed with the patient's arms down, compared to an RT planning CT with the patient's arms up, can generate difficulties in performing an accurate image registration for planning purposes. RTs knowledge of both anatomy, tumour progression/regression, response to treatment, potential treatment site and subsequent dosimetry are essential to performing relevant and usable IR. Other IR challenges are presented through limited field of view and the scan length of diagnostic imaging. In MRI, 2D acquisitions may include all relevant anatomy. However, if the slice thickness is greater than 2 mm, reconstructed planar views may have insufficient resolution to be usable, particularly if the diagnostic MRI also has a reduced field of view at the skin surface’. In addition, the primary acquisition plane may be sagittal or coronal. In these situations, registration may be achievable but more difficult to perform [17]. Obtaining at least one high resolution (< = 1 mm) 3D MRI sequence within a series is recommended to facilitate most accurate IR.

Image artefacts can diminish the usefulness of a dataset when near the region of interest. Where feasible, motion management should be used to reduce motion artefacts, for example, breath‐hold scans that correlate to a breath‐hold planning CT. Bladder and bowel filling changes between datasets can also render a secondary dataset unusable for radiation therapy planning purposes. Every effort to replicate organ filling and or voiding should be made where possible, further reiterating the value of strong relationships between diagnostic and radiation therapy departments to ensure acquired images are best fit for radiation therapy planning.

Per AAPM TG 132, the level of accuracy achieved for each IR performed in radiation therapy planning should be clearly documented [5]. Registration accuracy across an entire dataset may not be achievable and the addition of DIR can result in unrealistic anatomical changes that would affect tumour and OAR delineation. In the absence of repeat imaging, local alignment may prove useful; however, for larger targets, multiple IR may be required to allow accurate delineation of structures or dose assessment for organs at risk.

Robust quality assurance checks should be applied to IR with any uncertainties identified, clearly communicated and documented for all downstream processes such as target delineation and treatment planning. The level of accuracy required in IR will be determined by the end use of the registration. This should be considered when constructing QA processes.

## 4 ASMIRT Position on Clinical Use of IR in Treatment Planning

### 4.1 Target Definition

IR is routinely completed prior to target delineation but may also be performed during the process where further IR is requested. Optimally, the entire scan should be well matched; however, where tilt, rotation and anatomical changes are present, target and serial OAR location should be primarily considered for most accurate matching. Multiple RIR or DIR can be used to address changes in anatomy. The accuracy of the image alignment may impact margin selection for Planning Target Volumes.

In Australia, RTs are typically responsible for performing IR to ensure correct anatomical registration of targets and OAR, ensuring subsequent dosimetric assessment is accurate. An extensive understanding of human anatomy, tumour pathology and dosimetric principles is required to ensure that registrations presented to the treating RO are accurate for the context of their use, and performed according to instruction from and in consultation with the treating RO. RTs are responsible for providing, at minimum, a qualitative assessment of the accuracy and utility of the registration per AAPM TG 132 and should consider a quantitative assessment if it is deemed appropriate, such as in cases of extreme hypofractionation. Appropriate quantitative tolerances should be implemented by the multidisciplinary team, inclusive of RTs, dependent on the intended use of the image registration [5].

### 4.2 Auto Segmentation/Contour Mapping

Auto segmentation/contour mapping is the automated delineation of structures that are required to be included in the treatment planning process, namely target volumes and OARs. Traditionally, segmentation is performed manually and seen as a repetitive, time‐consuming task, often impacted by intra and inter‐user variation. IR, particularly DIR, can be used to increase the efficiency of contour definition/segmentation for the creation of the initial or subsequent treatment plans. Using IR can allow the rapid progression of adaptive workflows and the possibility of same‐day adaptation. Performing the segmentation can be triggered automatically or manually by a member of the RT planning team. Verification of the resultant contours is required to ensure accurate delineation and is the responsibility of the RO and the RT creating the treatment plan. RTs should maintain a good understanding of the limitations of the registration algorithms. Where resultant contours are manually adjusted, the underlying registration remains unchanged and therefore should not be used for purposes other than the original intention.

### 4.3 Dose Accumulation

Adaptive radiation therapy (ART) is used to optimise patients' treatment dose to maximise the therapeutic benefit. Adapting future dose to account for observed changes can be done in several ways. With the increasing availability, sophistication and integration of IR technology, it is possible to calculate the daily delivered dose to the patient on imaging acquired at the treatment unit. Volumetric imaging such as cone beam CT (CBCT) and MRI (as part of an integrated MR Linac platform), together with RIR based on the daily IGRT shifts determined by the treating RTs, is used for calculation and assessment of the daily delivered dose at the treatment location. DIR can be used to accumulate delivered dose and to auto segment transferred anatomical contours to assist in adaptation decision‐making and recording. The RT undertaking the treatment planning has the responsibility for checking that each step of the dose transfer and accumulation has been performed and recorded accurately, together with providing at minimum a qualitative assessment of that accuracy to the treating RO and should consider if a quantitative assessment is required. However, it must be noted that there can be in‐practice limitations, such as scan length and uncertainties associated with Hounsfield Unit conversions, when utilising CBCT for dose accumulation. RTs must take this into consideration, and if necessary, consider supporting methods such as electronic portal imaging device (EPID) dosimetry.

### 4.4 Adaptive and Previous Treatment

**FIGURE 1:**
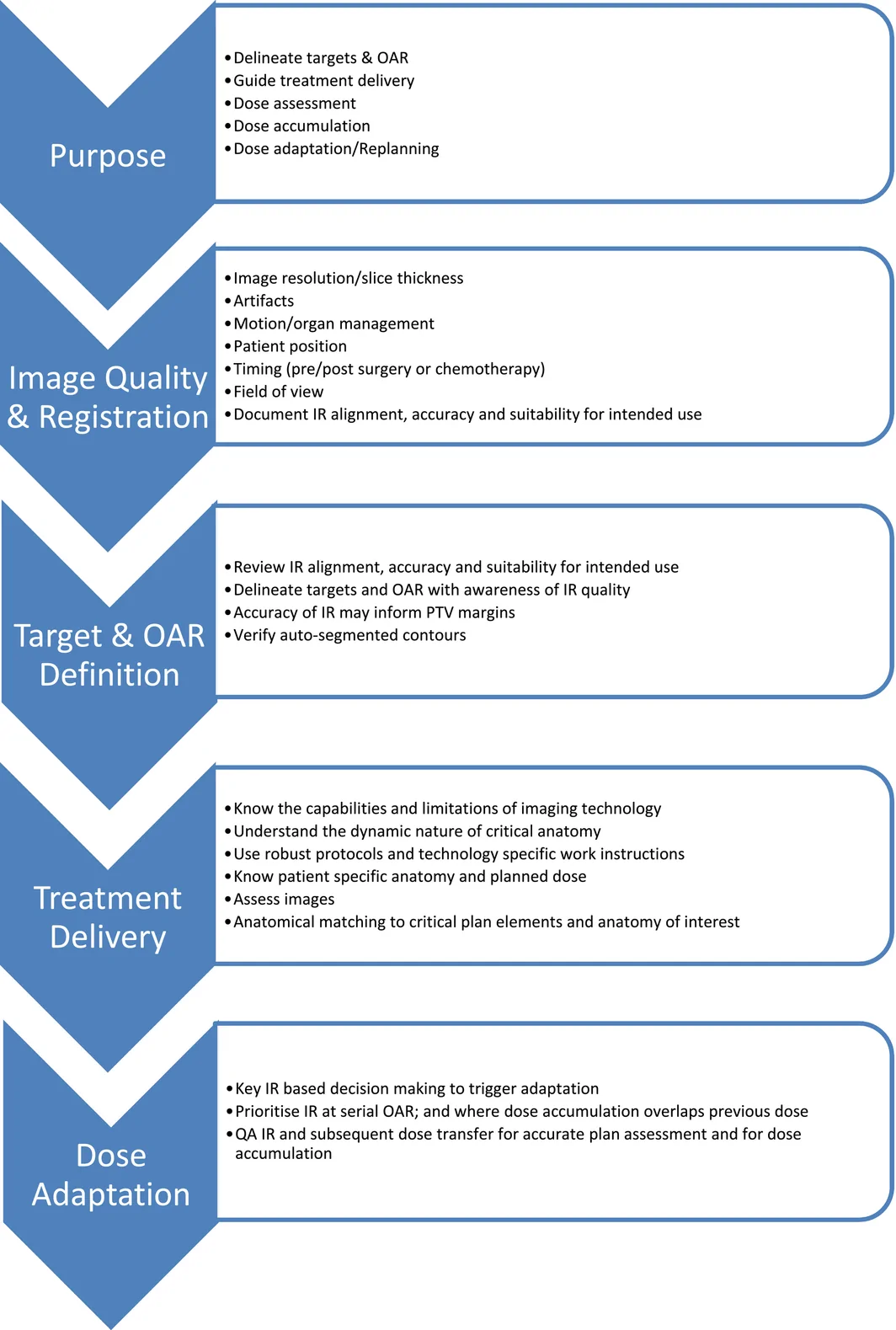
Summary of image registration process and considerations at each step.

It is common for patients to receive multiple courses of radiation therapy treatment. In some cases, there will be considerations regarding overlap doses, especially in relation to OARs. For this to be completed accurately, registration of the previous planning CT and current planning CT is required. For optimal registration and subsequent overlap doses, previous and current treatment positions should be replicated where possible and clinically appropriate. Registration of the two images should be completed (where possible) at the time of target delineation. This allows for timely consideration of potential overlap doses for the RO as they delineate target volumes and prescribe dose. Similar considerations to IR in the dosimetry process need to be considered, including the likelihood of a requirement for multiple registrations to accurately assess dose to OAR when RIR alone is used. Consultation with the RO may be necessary to determine the region of interest and tissue for the IR to be focused on. This is particularly pertinent in instances of reirradiation, where knowledge of cumulative doses to OAR and adjacent healthy tissue is critical to clinical decision‐making. Once again, DIR can be utilised to allow improved visualisation accuracy of the fusion between the images. Considerations need to be made about deformable dose review and the accuracy of dosimetry once DIR is utilised in this context. It is the responsibility of the RT to perform and document IR accuracy. ROs should review all IR to ensure it meets the needs for the overlap treatment dose review. Figure 1 provides a summary of the IR process and key considerations at each step.

The ASMIRT Radiation Therapist Scope of Practice document describes treatment planning and dosimetry skills including image manipulation and use of treatment planning software and associated tools for contouring, acting complementary to the required skillset detailed in this paper [18].

## 5 ASMIRT Position on Clinical Use of IR in Treatment Delivery

### 5.1 Online IR and IGRT

Daily online IGRT, either performed immediately preceding a treatment fraction or in real‐time during treatment fraction delivery, is a core responsibility of RTs. Although rigid IR forms the foundation of all IGRT, image critique and assessment is now more commonly requiring the interpretation of real time anatomical changes generated via multiple dynamic imaging and motion management platforms, for example, 4D CBCT, BrainLab ExacTrac (BrainLab, Germany), kilovoltage intrafraction monitoring (KIM), dynamic MRI sequences. When combined, each demands a thorough understanding of the anatomy from various image sources. Furthermore, the limitations of the technology utilised (e.g., couch, systematic errors and dosimetry) and the dynamic nature of critical anatomy (e.g., bladder filling, bowel gas, structure mobility such as small bowel) must also be well understood. Treatment intent, dose limiting structures, target margins, isodoses and other plan evaluation structures must also be well understood to inform appropriate clinical decision‐making, driven by robust clinical protocols and technology specific work instructions. Traffic light‐based imaging protocols have been used previously to assist RT's in clinical decision‐making [19]. The RT scope of practice requires RTs to make the final decision prior to treatment delivery, which in turn, requires thorough credentialing and supporting documentation for safe image guided, precision radiation therapy delivery [18].

### 5.2 Offline IR and IGRT

Subsequent assessment following robust IGRT protocols can be performed offline (after a delivered fraction or inter‐fraction) to inform decisions around continued suitability of the treatment plan. Offline IR assessment also helps identify systematic trends in patient positional correction and allows for thorough review of online image matching to ensure that appropriate decision‐making is happening at the time of treatment. This may require consultation with multidisciplinary colleagues to best inform the individual clinical needs of the patient.

### 5.3 ASMIRT Position on Clinical Use of IR in Adaptive Radiation Therapy

Adaptive Radiation Therapy (ART) is a closed loop treatment process allowing a patient's treatment plan to be modified to account for observed variation in patient measurement or circumstances [20, 21]. The goal of ART is to refine the treatment plan to improve the therapeutic ratio of the patient's treatment [22].

Modern ART relies heavily on the use of IR at each time point of IGRT, namely *offline* or *online*. Each of these IR processes involves key decision‐making, including decisions for future dose delivery such as a change to fractional target or OAR dose that will ultimately affect the patient's treatment outcome. The treating RT performs an important role in the ART workflow and together with the RO will decide when to trigger the adaptation of the treatment plan.

Timely ART relies on the use of IR to speed processes as described in the treatment planning section of this paper including target definition, OAR contouring and dose accumulation. IR can be used to transfer previously contoured volumes utilising either RIR or DIR and can also facilitate auto‐segmentation.

IR used for ART needs to follow the same recommendations for QA and reporting that IR used for planning or treatment delivery undergoes, no matter the time constraints that are present during these processes. Evaluation of both RIR and DIR is multifactorial and requires pre‐determined metrics‐based evaluation, as deemed pertinent to the individual clinical circumstance by the clinical team.

### 5.4 ASMIRT Position on the Roles and Responsibilities of RTs in IR

RT's, together with Radiation Oncology Medical Physicists and Radiation Oncologists, are responsible for performing and assessing IR within the Radiation Therapy department.

RTs are typically required to complete the following:

- Transfer of requested imaging into local IR systems
- Registration of multi‐modality diagnostic imaging with treatment planning CT for target delineation.
- Documentation of the suitability and quality of any IR performed for use in target and OAR delineation
- Perform and evaluate auto segmentation of planning CT, whereby contours are resultant from RIR or DIR.
- Registration of planning CT with imaging acquired at the treatment machine for IGRT.
- Registration of planning CT with required imaging for ART including dose accumulation.
- QA of IR and communication and documentation of registration accuracy, including limitations on subsequent use of registered images
- Protocol development and training in department‐specific use of IR
- Communicate imaging requirements with diagnostic imaging colleagues to enable optimal image registration where achievable.

### 5.5 Summary of Clinical Recommendations

1. Where reasonably possible, RTs should have ready access to PACS systems for retrieval of imaging.

2. Departments should make every attempt to follow nomenclature guidelines (e.g., TROG, AAPM).

3. RTs are responsible for verifying that the correct image datasets have been imported into the system for image registration (IR), and for performing and documenting any subsequent IR. This should be based on a thorough knowledge of anatomy, tumour pathology, and dosimetric principles, underpinned by undergraduate, clinical, and, where appropriate, postgraduate training.

4. QA of IR processes by RO, ROMP and RT should be embedded in individual department IR workflows.

5. For ART, RTs are responsible for performing and checking IR to ensure subsequent dose accumulation is accurate.

6. For reirradiation, multiple RIR or DIR of the same datasets may be required to evaluate dose accurately at the organ level.

7. RTs performing IGRT at the treatment interface should be appropriately credentialed for use of IR software and in IGRT practice, to ensure online critical decision‐making is performed efficiently and with full understanding of the treatment intent, patient‐specific dose limiting organs at risk, target margins and risks to inefficiency.

8. There should be a robust departmental protocol to guide IGRT practice.

9. RTs should perform offline IGRT review to systematically evaluate (i.e., via a set of predetermined metrics for each clinical plan) the continued suitability of a treatment plan, triggering ART as required.

10. Regardless of the purpose of the IR, for each IR the region aligned, the accuracy level and the recommended intended use should be clearly assessed and documented (per AAPM TG 132). This assessment should be provided for the treating RO to consider.

## Conflicts of Interest

The authors declare no conflicts of interest.

## Data Availability

Data sharing not applicable to this article as no datasets were generated or analysed during the current study.

## References

[1] D. van der Merwe , J. Van Dyk , B. Healy , et al., “Accuracy Requirements and Uncertainties in Radiotherapy: A Report of the International Atomic Energy Agency,” Acta Oncologica 56, no. 1 (2016): 1–6. 27846757 10.1080/0284186X.2016.1246801

[2] Radiation Oncology in Queensland: Indicators of Safe, Quality Cancer Care Delivered by Public and Private Services 2009‐2018, Cancer Alliance Queensland (2021), https://cancerallianceqld.health.qld.gov.au/media/1992/radiation‐oncology‐in‐qld‐2009‐2018.pdf.

[3] J. Barber , J. Yuen , M. Jameson , et al., “Deforming to Best Practice: Key Considerations for Deformable Image Registration in Radiotherapy,” Journal of Medical Radiation Sciences 67, no. 4 (2020): 318–332. 32741090 10.1002/jmrs.417 PMC7754021

[4] PSMA PET‐CT Accurately Detects Prostate Cancer Spread, Trial Shows, National Cancer Institute (2020), https://www.cancer.gov/news‐events/cancer‐currents‐blog/2020/prostate‐cancer‐psma‐pet‐ct‐metastasis#:%7E:text=Based%20on%20the%20imaging%2C%20PSMA,is%20present%20and%20not%20present.

[5] K. K. Brock , S. Mutic , T. R. McNutt , H. Li , and M. L. Kessler , “Use of Image Registration and Fusion Algorithms and Techniques in Radiotherapy: Report of the AAPM Radiation Therapy Committee Task Group No. 132,” Medical Physics 44 (2017): e43–e76. 28376237 10.1002/mp.12256

[6] J. Y. Luh , K. V. Albuquerque , C. Cheng , et al., “ACR‐ASTRO Practice Parameter for Image‐Guided Radiation Therapy (IGRT),” American Journal of Clinical Oncology 43, no. 7 (2020): 459–468. 32452841 10.1097/COC.0000000000000697

[7] X. S. Qi , K. V. Albuquerque , S. Bailey , et al., “Quality and Safety Considerations in Image Guided Radiation Therapy: An ASTRO Safety White Paper Update,” Practical Radiation Oncology 13, no. 2 (2023): 97–111. 36585312 10.1016/j.prro.2022.09.004

[8] On Target 2: Updated Guidance for Image‐Guided Radiotherapy. Royal College of Radiologists (2021), https://www.rcr.ac.uk/media/2pvoxjcp/rcr_publication‐on‐target‐2‐updated‐guidance‐for‐image‐guided‐radiotherapy.pdf. 10.1016/j.clon.2021.10.002 34728132

[9] P. J. Keall , G. S. Mageras , J. M. Balter , et al., “The Management of Respiratory Motion in Radiation Oncology Report of AAPM Task Group 76,” Medical Physics 33, no. 10 (2006): 3583–3976. 17089851 10.1118/1.2349696

[10] J. P. Schiff , T. Zhao , Y. Huang , et al., “Simulation‐Free Radiation Therapy: An Emerging Form of Treatment Planning to Expedite Plan Generation for Patients Receiving Palliative Radiation Therapy,” Advances in Radiation Oncology 8, no. 1 (2022): 101091. 36304132 10.1016/j.adro.2022.101091 PMC9594122

[11] T. Schuler , M. Back , G. Hruby , et al., “Introducing Computed Tomography Simulation‐Free and Electronic Patient‐Reported Outcomes‐Monitored Palliative Radiation Therapy Into Routine Care: Clinical Outcomes and Implementation Experience,” Advances in Radiation Oncology 6, no. 2 (2020): 100632. 33851063 10.1016/j.adro.2020.100632 PMC8039552

[12] S. Wong , S. Roderick , A. Kejda , et al., “Diagnostic Computed Tomography Enabled Planning for Palliative Radiation Therapy: Removing the Need for a Planning Computed Tomography Scan,” Practical Radiation Oncology 11, no. 2 (2021): e146–e153. 33186781 10.1016/j.prro.2020.10.010

[13] S. Larjavaara , S. Strengell , T. Seppälä , et al., “Palliative Intensity Modulated Radiotherapy of Bone Metastases Based on Diagnostic Instead of Planning Computed Tomography Scans,” Physics and Imaging in Radiation Oncology 27 (2023): 100456. 37720465 10.1016/j.phro.2023.100456 PMC10500021

[14] TROG New Techniques Committee , TROG Standardised Naming and Contouring Guidelines (2021).

[15] C. S. Mayo , J. M. Moran , W. Bosch , et al., “American Association of Physicists in Medicine Task Group 263: Standardizing Nomenclatures in Radiation Oncology,” International Journal of Radiation Oncology, Biology, Physics 100, no. 4 (2018): 1057–1066. 29485047 10.1016/j.ijrobp.2017.12.013 PMC7437157

[16] N. Lowther , R. Louwe , J. Yuen , et al., “MIRSIG Position Paper: The Use of Image Registration and Fusion Algorithms in Radiotherapy,” Physical and Engineering Sciences in Medicine 45, no. 2 (2022): 421–428. 35522369 10.1007/s13246-022-01125-3 PMC9239966

[17] A. McWilliam , B. Rowland , and M. van Herk , “The Challenges of Using MRI During Radiotherapy,” Clinical Oncology 30, no. 11 (2018): 680–685. 30197096 10.1016/j.clon.2018.08.004

[18] Radiation Therapist Scope of Practice. Australian Society of Medical Imaging and Radiation Therapy (2017), https://asmirt.org/wp‐content/uploads/2024/04/RT‐scope‐of‐practice.pdf.

[19] M. Buijs , F. Pos , M. Frantzen‐Steneker , et al., “Take Action Protocol: A Radiation Therapist Led Approach to Act on Anatomical Changes Seen on CBCT,” Technology Innovations in Patient Support and Radiation Oncology 17 (2021): 71–77. 10.1016/j.tipsro.2020.12.001 PMC8110944 34007910

[20] D. Yan , F. Vicini , J. Wong , and A. Martinez , “Adaptive Radiation Therapy,” Physics in Medicine and Biology 42, no. 1 (1997): 123–132, 10.1088/0031-9155/42/1/008. 9015813

[21] P. J. Keall , A. Hsu , and L. Xing , “12—Image‐Guided Adaptive Radiotherapy,” in Leibel and Phillips Textbook of Radiation Oncology, 3rd ed., ed. R. T. Hoppe , T. L. Phillips , and M. Roach (W.B. Saunders, 2010), 213–223.

[22] K. K. Brock , “Adaptive Radiotherapy: Moving Into the Future,” Seminars in Radiation Oncology 29, no. 3 (2019): 181–184, 10.1016/j.semradonc.2019.02.011. 31027635 PMC7219982

